# Using egg production longitudinal recording to study the genetic background of resilience in purebred and crossbred laying hens

**DOI:** 10.1186/s12711-022-00716-8

**Published:** 2022-04-20

**Authors:** Nicolas Bedere, Tom V. L. Berghof, Katrijn Peeters, Marie-Hélène Pinard-van der Laan, Jeroen Visscher, Ingrid David, Han A. Mulder

**Affiliations:** 1 PEGASE, INRAE, Institut Agro, 35590 Saint Gilles, France; 2 Wageningen University & Research Animal Breeding & Genomics, P.O. Box 338, 6700 AH Wageningen, The Netherlands; 3 Reproductive Biotechnology, TUM School of Life Sciences, Technical University of Munich, Liesel‑Beckmann‑Strasse 1, 85354 Freising, Germany; 4 Hendrix Genetics B.V., P.O. Box 114, 5830 AC Boxmeer, The Netherlands; 5 Université Paris-Saclay, INRAE, AgroParisTech, GABI, 78350 Jouy-en-Josas, France; 6 GenPhySE, Université de Toulouse, INRAE, ENVT, 31320 Castanet Tolosan, France

## Abstract

**Background:**

There is growing interest in using genetic selection to obtain more resilient farm animals (i.e. that are minimally affected by disturbances or rapidly recover from them). The aims of this study were to: (i) estimate the genetic parameters of resilience indicator traits based on egg production data, (ii) assess whether these traits are genetically correlated in purebreds and crossbreds, and (iii) assess the genetic correlations of these traits with egg production (EP) as total number of eggs between 25 and 83 weeks. Purebred hens (33,825 from a White Leghorn (WA) line and 34,397 from a Rhode Island (BD) line were housed in individual cages, while crossbred hens were housed in collective cages of 6 to 8 paternal half-sibs (12,852 WA and 3898 BD crossbred groups, where the name of the group refers to the line used as the sire). Deviations of a hen’s weekly egg production from the average of the corresponding batch were calculated. Resilience indicator traits investigated were the natural logarithm of the variance (LNVAR), the skewness (SKEW), and the lag-one autocorrelation (AUTO-R) of these deviations.

**Results:**

In both purebred lines, EP was estimated to be lowly heritable (WA: 0.11 and BD: 0.12). Resilience indicators were also estimated to be lowly heritable in both lines (LNVAR: 0.10 and 0.12, SKEW: 0.04 and 0.02, AUTO-R: 0.06 and 0.08 in WA and BD, respectively). In both crossbred groups, EP, AUTO-R, and SKEW were estimated to be less heritable than in purebreds (EP: $h^{2}$ ≤ 0.07; and resilience indicator traits: $h^{2}$ ≤ 0.03), while LNVAR had an $h^{2}$ estimate that was similar to or higher in crossbreds ($h^{2}$ ranged from 0.13 to 0.21) than in purebreds. In both purebreds and crossbreds, resilience indicator traits were estimated to have favorable genetic correlations with EP and between each other. For all traits and in both lines, estimates of genetic correlations between purebreds and crossbreds ($r_{pc}$) differed from 1 and ranged from 0.16 to 0.63.

**Conclusions:**

These results show that selection for resilience based on EP data can be considered in breeding programs for layers. Genetic improvement of resilience in crossbreds can be achieved by using information on purebreds, but would be greatly enhanced by the integration of information on crossbreds in breeding programs.

**Supplementary Information:**

The online version contains supplementary material available at 10.1186/s12711-022-00716-8.

## Background

Over the past 60 years, breeding programs have resulted in very productive farm animals, at the expense of functional traits related to fitness (longevity, health, reproduction, etc.) [1, 2]. These trade-offs between biological functions can be explained by the “resource allocation theory”: when two biological processes share the same resources, they are competing in situations of limited resources [3]. Situations of limited resources are more likely to occur nowadays because of the increasing competition for natural resources with other animals, or with humans [4]. Moreover, situations of additional energy expenses are more frequent because of negative social interactions due to group housing [5] and increasing risks of diseases, temperature fluctuations, for instance with outdoor access and reduced use of preventive antibiotics. Therefore, there is growing interest to select for improved resilience in farm animals, although improvement of general resilience is not commonly implemented in breeding programs [6].

Resilience is defined as “the capacity of an animal to be minimally affected by disturbances or to rapidly return to the state pertained before exposure to a disturbance” [5, 6]. One reason why resilience has not yet been implemented in breeding programs is due to the lack of selection criteria and recording tools. Opportunities to construct selection criteria for resilience lie in the collection of repeated information per individual or per family [7]. Indeed, the calculation of resilience indicator traits from longitudinal data is possible in various species [8]. Animals that are exposed to a disturbance are known to show a different biological response than expected without a disturbance (e.g. growth, feed intake, milk production) [5, 9, 10]. Thus, the most studied resilience indicators are derived from deviations between the observed and expected performance (such as the variance of the deviations) or directly from longitudinal analyses (such as the slope of a reaction norm). These indicators are known to be associated with health-status and periods of perturbations [11–14]. Only a few studies have investigated the genetic parameters of resilience indicator traits derived from longitudinal data in farm animals: dairy cows [15, 16], pigs [17], and layers [18]. Resilience indicator traits based on longitudinal recording of milk production [15, 16], feed intake [17], or body weight [18] have been shown to be heritable and have favorable genetic correlations with health-related traits.

Previous studies in laying hens have investigated resilience indicators that were derived from body weight [18], egg weight [19], or eggshell color [20]. Because these traits were monitored only at specific time points, the number of records was very small in these studies (3 to 8 records during the production cycle per hen). However, breeders record the number of eggs laid daily, at the individual level in nucleus farms or at a group cage level in recurrent test farms. Resilience indicators that are derived from daily data on egg production have not been investigated but may hold potential to implement selection on resilience in breeding programs for layers. The breeding goal of poultry breeding companies is to genetically improve multiple traits in commercial hens, often a 3- or 4-way cross. To do that, they use a pyramidal breeding program, i.e., they perform genetic selection on purebreds, to genetically improve performance of crossbreds. Records on individual performance are required in the nucleus to select birds with the desired characteristics. Consequently, pure line birds are individually housed to enable individual identification and recording of own performance (e.g. individual egg traits). Two-way crossbred animals are placed in recurrent field tests (RT), in collective cages (i.e. housed in groups) to mimic commercial farm conditions. Crossbred phenotypes are recorded on the group level. These crossbred animals are pedigreed to allow the estimation of genetic parameters and breeding values of purebreds. The purebred–crossbred genetic correlation for a trait is often lower than 1 [21, 22], which implies that crossbred information is valuable to estimate breeding values of purebred birds for crossbred performance [23, 24].

Our objectives were: (i) to study the genetic parameters of resilience indicator traits that are derived from longitudinal egg production data, (ii) to assess whether these traits are genetically correlated in purebreds and crossbreds (purebred–crossbred correlation), and (iii) to assess the genetic correlations of these traits with egg production. To our knowledge, this is the first time that egg production longitudinal recording is used to study resilience in laying hens. The purebred–crossbred correlation determines whether selection for improved resilience in purebreds will result in improved resilience in crossbreds. It also determines the added value of collecting crossbred information: the lower the purebred–crossbred correlation, the higher the value of crossbred information when crossbred performance is the breeding goal [23, 24]. Estimates of genetic correlations with other traits will provide knowledge about the overlap between the genetic background of the traits and practical insights about the integration of resilience traits in breeding programs.

## Methods

### Study populations

In this study, two purebred lines from Hendrix Genetics were studied: a White Leghorn line, called WA, and a Rhode Island line, called BD. The WA purebreds were individually housed on a nucleus farm in The Netherlands (33,825 layers) and distributed among 13 batches from 2012 to 2017. The BD purebreds were individually housed on a nucleus farm in France (34,397 layers) and distributed among 14 batches from 2012 to 2017 (further details about the study design are in Additional file 1: Tables S1 and S2). Recurrent field tests were carried out in Canada (CA) and The Netherlands (NL) by crossing purebred sires from the WA or the BD line with dams from five and seven other lines, respectively. The crossbreds were housed in cages, in groups of 6 to 8 paternal half-sibs. All crossbreds were beak-trimmed but the purebreds were not. The final dataset included records on 12,852 groups of WA crossbreds (6154 records on cages of 6 hens and 6698 records on cages of 7 hens) distributed among four CA farms and six NL farms and 17 batches, and records on 3898 groups of BD crossbreds (2428 records on cages of 7 hens and 1470 records on cages of 8 hens) distributed among one CA farm and four NL farms and nine batches. For crossbred RT, 71 and 29% of the sires used in the WA and BD lines, respectively, had offspring in both countries (CA and NL). The purebred and crossbred populations consisted mostly of half-sib families with, on average, 50 offspring per sire in purebreds and 70 offspring per sire in crossbreds. The pedigree for each recorded individual was traced back between 4 and 16 generations (paternal only for crossbreds).

### Egg production recording

Eggs were collected several times a week (with the interval between two collections ranging from 1 to 4 days). Therefore, the raw data consisted of dates and number of eggs collected. Egg production was recorded individually for purebreds and as a pooled average for a cage for crossbreds, i.e., the total number of eggs produced during a particular period was divided by the average number of hens alive in the cage during that period. Observations for individual purebred hens with two eggs per day were not discarded from the analyses, since the first egg could have been laid after collection time on the day before and the second egg could have been laid before collection time on the day of recording. Observations of three or more eggs per day were discarded from the analyses, because this is not biologically possible, even with different collection times in successive days. Daily egg production was estimated for every day between two recording dates by dividing the total number of eggs by the number of days in the period. These daily averages were summed over 7 days to obtain the weekly egg production (total number of eggs laid per week per hen). The trait egg production (EP) was defined as the total number of eggs laid per hen between 25 and 83 weeks of age.

### Resilience indicator traits based on egg production

The longitudinal egg production data enabled the computation of the types of resilience indicators based on deviations from the average performance of a contemporary group that had been used for other species in other studies [6, 16, 18]. The average weekly number of eggs of the batch was calculated as the total number of eggs divided by the number of hens alive in the batch and used to calculate deviations of individual weekly EP records from the batch average. Resilience indicator traits evaluated were the natural logarithm of the variance (LNVAR) of an individual’s or group’s deviations, the skewness of the distribution of the deviations (SKEW), and the lag-one autocorrelation (AUTO-R) of the deviations. These three traits are complementary [6]: a higher LNVAR is an indication of poor resilience of an animal to a perturbation, while resilient animals show a smaller range of deviations from the expected performance and therefore a smaller LNVAR; a more negative SKEW means that the animal shows more negative than positive deviations or that the animal experiences severe drops from the expected performance, which is an indication of poor resilience; and the closer AUTO-R is to 1, the more two subsequent deviations are alike, which is interpreted as a slower recovery from the perturbation and thus an indication of poor resilience. Observations for the resilience traits were discarded if they were outside the mean plus or minus four times the standard deviation within populations (combination of lines and purebreds/crossbreds separately). However, only the observation for the particular resilience indicator trait was discarded, i.e., observations for all other resilience indicators and egg production were kept. This resulted in the removal of about 4 and 8% of the raw data for each resilience indicator trait in the WA and BD lines, respectively.

Data handling and graphs were performed in base R [25]. Resilience indicator traits were calculated in R, i.e. LNVAR with the $log$ and $var$ functions, AUTO-R with the $acf$ function, and SKEW with the $skewness$ function of the $e1071$ package [26].

### Estimation of variance components for the same trait between purebreds and crossbreds

Within sire lines, variance components were estimated using bivariate analyses, considering records of the purebreds and records of the crossbreds from the same sires’ line as two different traits, to allow estimation of the purebred–crossbred correlation. The model used for purebred phenotypes was the following animal model:

$$y_{ijl } = \mu + HatchLoc_{i} + SurvClass_{j} + a_{l} + e_{ijl} ,$$

where $y$ is the observed phenotype (EP, LNVAR, SKEW, AUTO-R) of the $l$th purebred hen; $HatchLoc$ is the fixed effect for the $i$th combination of hatch week, barn, corridor, and row of the cage; $SurvClass$ is the fixed effect of the $j$th class of lifespan duration, with classes comprising intervals of four weeks, from the shortest lifespan observed to the longest (24 to 112 weeks of age), which allowed for the potential effect of survival on phenotypes to be accounted for without assuming a linear relationship between survival and the phenotype (as a covariate would do); $a$ is the random additive genetic effect of the $l$th purebred layer; and $e$ is the random residual effect.

The model used for crossbred phenotypes was the following sire model:

$$y_{ijkl} = \mu + HatchLoc_{i} + SurvClass_{j} + MaternalLine_{k} + s_{l} + e_{ijkl} ,$$

where $y$ is the mean observed phenotype (EP, LNVAR, SKEW, or AUTO-R) from a cage of crossbred halfsib hens from the $l$th sire; $HatchLoc$ and $SurvClass$ are the same fixed effects as in the animal model; $MaternalLine$ is the fixed effect of the $k$th maternal line of the crossbred offspring; $s$ is the random sire genetic effect of the $l$th purebred sire on phenotype of the crossbred offspring; and $e$ is the random residual effect.

The animal effects for the purebred phenotypes and the sire effects for the crossbred phenotypes were assumed to be bivariate normally distributed as:

$$\left[ {\begin{array}{*{20}c} {{\mathbf{a}}_{{\text{p}}} } \\ {{\mathbf{s}}_{{\text{c}}} } \\ \end{array} } \right]\sim MVN\left( {\left( {\begin{array}{*{20}c} 0 \\ 0 \\ \end{array} } \right), {\mathbf{A}} \times \left[ {\begin{array}{*{20}c} {\sigma_{{a_{p} }}^{2} } \\ {\sigma_{{a_{p} s_{c} }} } \\ \end{array} \begin{array}{*{20}c} { \sigma_{{a_{p} s_{c} }} } \\ { \sigma_{{s_{c} }}^{2} } \\ \end{array} } \right]} \right)\;{\text{with}}\;\sigma_{{s_{c} }}^{2} = \frac{1}{4}\sigma_{{a_{c} }}^{2} ,$$

where ${\mathbf{a}}_{{\text{p}}}$ is the vector of breeding values for purebred performance and ${\mathbf{s}}_{{\text{c}}}$ is the vector of sire genetic effects for crossbred performance ($p$ indicates purebreds and $c$ indicates crossbreds; ${\mathbf{A}}$ is the additive genetic relationship matrix; $\sigma_{{a_{p} }}^{2}$ is the additive genetic variance for purebred performance (animal model); $\sigma_{{s_{c} }}^{2}$ is the sire genetic variance for crossbred performance (sire model); $\sigma_{{a_{c} }}^{2}$ is the additive genetic variance for crossbred performance; and $\sigma_{{a_{p} s_{c} }}$ is the genetic covariance between purebred and crossbred performance (note: $\sigma_{{a_{p} s_{c} }} = r_{pc} \times \sigma_{{a_{p} }} \times \sigma_{{s_{c} }} = \frac{1}{2} \times r_{pc} \times \sigma_{{a_{p} }} \times \sigma_{{a_{c} }}$, where $r_{pc}$ is the purebred–crossbred genetic correlation). Heterogeneity of residual variance was modeled with a single residual variance for individuals for purebred phenotypes and a separate residual variance for each cage size for crossbred phenotypes (cages of 6 or 7 hens in the WA line and cages of 7 or 8 hens in the BD line). We chose this approach because we observed that the residual variance varied with cage size. The residual effects were assumed to be distributed as:

$$\left[ {\begin{array}{*{20}c} {{\mathbf{e}}_{{\text{p}}} } \\ {{\mathbf{e}}_{{{\text{c}}6}} } \\ {{\mathbf{e}}_{{{\text{c}}7}} } \\ \end{array} } \right]\sim MVN\left( {\left( {\begin{array}{*{20}c} 0 \\ 0 \\ 0 \\ \end{array} } \right),\; {\mathbf{I}} \times \left[ {\begin{array}{*{20}c} {\sigma_{{e_{p} }}^{2} } \\ 0 \\ 0 \\ \end{array} \begin{array}{*{20}c} 0 \\ {\sigma_{{e_{c6} }}^{2} } \\ 0 \\ \end{array} \begin{array}{*{20}c} 0 \\ 0 \\ {\sigma_{{e_{c7} }}^{2} } \\ \end{array} } \right]} \right) \;{\text{for}}\;{\text{the}}\;{\text{WA}}\;{\text{line}},$$

$$\left[ {\begin{array}{*{20}c} {{\mathbf{e}}_{{\text{p}}} } \\ {{\mathbf{e}}_{{{\text{c}}7}} } \\ {{\mathbf{e}}_{{{\text{c}}8}} } \\ \end{array} } \right]\sim MVN\left( {\left( {\begin{array}{*{20}c} 0 \\ 0 \\ 0 \\ \end{array} } \right),\;{\mathbf{I}} \times \left[ {\begin{array}{*{20}c} {\sigma_{{e_{p} }}^{2} } \\ 0 \\ 0 \\ \end{array} \begin{array}{*{20}c} 0 \\ {\sigma_{{e_{c7} }}^{2} } \\ 0 \\ \end{array} \begin{array}{*{20}c} 0 \\ 0 \\ {\sigma_{{e_{c8} }}^{2} } \\ \end{array} } \right]} \right)\;{\text{for}}\;{\text{the}}\;{\text{BD}}\;{\text{line}},$$

where ${\mathbf{I}}$ is the identity matrix, $\sigma_{{e_{p} }}^{2}$ is the residual variance for purebreds, $\sigma_{{e_{c} }}^{2}$ is the residual variance for crossbreds in cages of 6, 7, or 8 grouped hens. A residual covariance between purebred and crossbred performance was not modeled because hens were present in only one of the three environments.

### Estimation of variance components for different traits within purebreds and crossbreds

Bivariate analyses were used to study the genetic relationship between traits, using the same models presented above (animal model for purebred phenotypes and sire model for crossbred phenotypes). Within each purebred population and for each pair of traits, the genetic effects were assumed to be distributed as:

$$\left[ {\begin{array}{*{20}c} {{\mathbf{a}}_{{{\text{trait}}.1}} } \\ {{\mathbf{a}}_{{{\text{trait}}.2}} } \\ \end{array} } \right]\sim MVN\left( {\left( {\begin{array}{*{20}c} 0 \\ 0 \\ \end{array} } \right), {\mathbf{A}} \times \left[ {\begin{array}{*{20}c} {\sigma_{{a_{trait.1} }}^{2} } \\ {\sigma_{{a_{trait.1,trait.2} }} } \\ \end{array} \begin{array}{*{20}c} { \sigma_{{a_{trait.1,trait.2} }} } \\ { \sigma_{{a_{trait.2} }}^{2} } \\ \end{array} } \right]} \right),$$

where the subscripts $trait.1$ and $trait.2$ indicate the two traits analyzed, ${\mathbf{A}}$ is the additive genetic relationship matrix, $\sigma_{{a_{trait} }}^{2}$ is the additive genetic variance, $\sigma_{{a_{trait.1,trait.2} }}$ is the genetic covariance between the two traits.

Within each crossbred population and for each pair of traits, the genetic effects were assumed to be distributed as:

$$\left[ {\begin{array}{*{20}c} {{\mathbf{s}}_{{{\text{trait}}.1}} } \\ {{\mathbf{s}}_{{{\text{trait}}.2}} } \\ \end{array} } \right]\sim MVN\left( {\left( {\begin{array}{*{20}c} 0 \\ 0 \\ \end{array} } \right), {\mathbf{A}} \times \left[ {\begin{array}{*{20}c} {\sigma_{{s_{trait.1} }}^{2} } \\ {\sigma_{{s_{trait.1,trait.2} }} } \\ \end{array} \begin{array}{*{20}c} { \sigma_{{s_{trait.1,trait.2} }} } \\ { \sigma_{{s_{trait.2} }}^{2} } \\ \end{array} } \right]} \right)\;{\text{with}}\;\sigma_{{s_{c} }}^{2} = \frac{1}{4}\sigma_{{a_{c} }}^{2} .$$

The residual effects were assumed to be distributed as:

$$\left[ {\begin{array}{*{20}c} {{\mathbf{e}}_{{{\text{trait}}.1}} } \\ {{\mathbf{e}}_{{{\text{trait}}.2}} } \\ \end{array} } \right]\sim MVN\left( {\left( {\begin{array}{*{20}c} 0 \\ 0 \\ \end{array} } \right), \;{\mathbf{I}} \times \left[ {\begin{array}{*{20}c} {\sigma_{{e_{trait.1} }}^{2} } \\ {\sigma_{{e_{trait.1,trait.2} }} } \\ \end{array} \begin{array}{*{20}c} { \sigma_{{e_{trait.1,trait.2} }} } \\ { \sigma_{{e_{trait.2} }}^{2} } \\ \end{array} } \right]} \right),$$

where ${\mathbf{I}}$ is the identity matrix, $\sigma_{e}^{2}$ is the residual variance for each trait and $\sigma_{{e_{trait.1,trait.2} }}$ is the residual covariance between the two traits, because in this case the two traits were measured on the same individuals. Each cage size was analyzed separately (2 sizes each for WA and BD crossbreds), because of the different residual variances. Variance component estimation was performed with the statistical software ASReml 4.2 [27].

### Heritability estimation

For purebred phenotypes, heritability estimates were calculated from the estimates of variance components obtained with the preceding models as:

$$h_{p}^{2} = \frac{{\sigma_{{a_{p} }}^{2} }}{{\sigma_{{a_{p} }}^{2} + \sigma_{{e_{p} }}^{2} }}.$$

For crossbred phenotypes, the calculation of the heritability needs to account for (i) the fact that the phenotypes are the average per cage of the pooled performance from all the layers in the cage and (ii) for the half-sib relationships between the grouped layers. The formula to scale heritability estimated with pooled data to an individual-based heritability was established in previous studies [28–30], as shown in Additional file 2, and was as follows (applied separately to estimates of variance components for each cage size):

$$h_{c \cdot n}^{2} = \frac{{4\sigma_{{s_{c} }}^{2} }}{{\sigma_{{P_{c}^{*} }}^{2} }}\;{\text{with}}\;\sigma_{{P_{c}^{*} }}^{2} = \sigma_{{s_{c} }}^{2} + n\sigma_{{e_{c \cdot n} }}^{2} ,$$

where $n$ is the initial number of hens grouped in the cage (ranging from 6 to 8), $4\sigma_{{s_{c} }}^{2}$ is the additive genetic variance for crossbreds; and $\sigma_{{e_{c} }}^{2}$ is the residual variance for crossbreds in cages of 6, 7, or 8 grouped hens. However, because, mathematically, the group phenotype for LNVAR is not the mean of individual records of LNVAR (see the details of the transformations in Additional file 2), its heritability was estimated as:

$$h_{c \cdot n}^{2} = \frac{{4\sigma_{{s_{c} }}^{2} }}{{\sigma_{{s_{c} }}^{2} + \sigma_{{e_{c \cdot n} }}^{2} }}.$$

### Significance testing

Likelihood-ratio (LR) tests were performed to test if the estimates of heritability were statistically significantly different from 0 or if the genetic correlations were different from 0 or 1. The LR was calculated as:

$$LR = - 2\left( {\ell_{full} - \ell_{constrained} } \right),$$

where $\ell_{full}$ is the likelihood of the univariate model for estimation of heritabilities and of the bivariate model for estimation of genetic correlations between traits, and $\ell_{constrained}$ is the likelihood of the corresponding constrained model. To test whether heritability estimates were statistically different from 0, the constrained models were identical to the univariate full models, except that they were fitted without the random genetic effect. To test whether the genetic correlations were statistically significantly different from 0 or 1, the constrained models were identical to the bivariate full models, except that the genetic covariance was set to 0 or 1 (using the $rr1$ structure for the genetic effects in ASReml 4.2 [27]). Assuming that $LR\sim \chi_{(n)}^{2}$, where $n$ is the reduction of the number of degrees of freedom between the compared models, the p-value can be retrieved from the chi-squared distribution. Note that when the comparison of models corresponds to a test of a parameter on the boundary of parameter space (test of heritability equal to 0 or correlation equal to 1), the distribution of this test statistic under the null hypothesis is a 50:50 mixture of $\chi_{0}^{2}$ and $\chi_{1}^{2}$ distributions (i.e. the threshold value for $\propto = 0.05$ is $\chi^{2} = 2.706$) [31].

## Results

### Observed egg production and resilience indicator traits

The two purebred lines were high-producing layers. The observed mean EP (between 25 and 83 weeks of age) was 371 and 347 eggs in the WA and BD purebreds, respectively (Table 1). Compared to the purebreds, the crossbreds had a similar EP, a lower LNVAR (i.e. smaller the fluctuations), a higher SKEW (i.e. a less symmetric distribution of the deviations), and a comparable AUTO-R (a higher AUTO-R corresponds to two subsequent deviations being more alike). The standard deviations were large for all traits (phenotypic coefficient of variation of about 10% for most traits), although they were smaller in crossbreds than in purebreds. It is important to keep in mind that crossbreds were housed in groups while purebreds were housed individually and averages typically have a lower variance than individual records.

**Table 1:** Phenotypic means and standard deviations (SD) of the four analyzed traits in purebreds and crossbreds from the WA and BD lines

Traits	Type	WA line		BD line	
		Mean	SD	Mean	SD
EP	Purebred	371	51	347	69
	Crossbred	368	24	341	36
LNVAR	Purebred	− 0.81	0.94	− 0.33	1.09
	Crossbred	− 2.32	0.73	− 1.84	0.64
SKEW	Purebred	− 1.51	1.03	− 1.20	1.15
	Crossbred	− 0.58	0.65	− 0.40	0.63
AUTO-R	Purebred	0.21	0.24	0.36	0.29
	Crossbred	0.19	0.27	0.34	0.23

*EP* total number of eggs laid per layer between 25 and 83 weeks of age, *LNVAR* natural logarithm of the variance of the deviations between the observed phenotype and the average of the batch, *SKEW* skewness of the distribution of these deviations, *AUTO-R* lag-one autocorrelation of these deviations

### Genetic parameters of egg production and resilience indicator traits

For purebreds, EP were estimated to be moderately heritable (WA: 0.11 in Table 2; BD: 0.12 in Table 3), while resilience indicators traits were estimated to be low to moderately heritable, depending on the trait. Heritability estimates were equal to 0.10 and 0.12 for LNVAR, 0.04 and 0.02 for SKEW, and 0.06 and 0.08 for AUTO-R in the WA and BD lines, respectively.

**Table 2:** Estimates of genetic parameters with their standard error in parentheses for pure- and crossbred layers of the WA line

	EP	LNVAR	SKEW	AUTO-R
Genetic variance				
$\sigma_{{a_{p} }}^{2}$	107 (10)	0.065 (0.006)	0.037 (0.006)	0.003 (< 0.001)
$\sigma_{{a_{c} }}^{2}$	91 (9)	0.046 (0.008)	0.037 (0.006)	0.003 (< 0.001)
Residual variance				
$\sigma_{{e_{p} }}^{2}$	873 (10)	0.609 (0.006)	0.920 (0.008)	0.047 (< 0.001)
$\sigma_{{e_{c \cdot 6} }}^{2}$	214 (4)	0.343 (0.007)	0.399 (0.008)	0.060 (0.001)
$\sigma_{{e_{c \cdot 7} }}^{2}$	242 (5)	0.336 (0.006)	0.382 (0.007)	0.065 (0.001)
Total variance				
$\sigma_{{P_{p} }}^{2}$	980 (9)	0.675 (0.006)	0.957 (0.008)	0.050 (< 0.001)
$\sigma_{{P_{c \cdot 6}^{*} }}^{2}$	1307 (25)	0.356 (0.007)	2.400 (0.045)	0.361 (0.007)
$\sigma_{{P_{c \cdot 7}^{*} }}^{2}$	1711 (31)	0.346 (0.006)	2.681 (0.048)	0.456 (0.008)
Genetic parameters				
$h_{P}^{2}$	0.11 (0.01)	0.10 (0.01)	0.04 (0.01)	0.06 (0.01)
$h_{c \cdot 6}^{2}$	0.07 (0.01)	0.13 (0.02)	0.006 (0.002)	0.02 (< 0.01)
$h_{c \cdot 7}^{2}$	0.05 (0.01)			0.01 (< 0.01)
$r_{pc}$	0.31 (0.10)	0.16 (0.13)	0.20 (0.23)	0.63 (0.14)

The variances reported are the variances estimated by the bivariate analyses with the same trait in purebreds and crossbreds analyzed jointly

*EP* total number of eggs laid per layer between 25 and 83 weeks of age, *LNVAR* natural logarithm of the variance of the deviations between the observed phenotype and the average of the batch, *SKEW* skewness of the distribution of these deviations, *AUTO-R* lag-one autocorrelation of these deviations

$\sigma_{{a_{p} }}^{2}$: additive genetic variances for purebreds; $\sigma_{{a_{c} }}^{2}$: additive genetic variances for crossbreds; $\sigma_{{e_{p} }}^{2}$: residual variances for purebreds; $\sigma_{{e_{c \cdot n} }}^{2}$: residual variances for r crossbreds, where $n$ is the number of hens in the cage; $\sigma_{{P_{p} }}^{2}$: total variance for purebreds; $\sigma_{{P_{c \cdot n}^{*} }}^{2}$: total variance for crossbreds; $h_{P}^{2}$: heritability estimates for purebreds; $h_{c \cdot n}^{2}$: heritability estimates for crossbreds; $r_{pc}$: genetic correlation between purebred and crossbreds

**Table 3:** Estimates of genetic parameters estimates with their standard error in parentheses for pure- and crossbred layers of the BD line

	EP	LNVAR	SKEW	AUTO-R
Genetic variance				
$\sigma_{{a_{p} }}^{2}$	325 (29)	0.137 (0.012)	0.027 (0.006)	0.006 (0.001)
$\sigma_{{a_{c} }}^{2}$	96 (19)	0.055 (0.015)	0.016 (0.013)	0.009 (0.002)
Residual variance				
$\sigma_{{e_{p} }}^{2}$	2400 (27)	1.022 (0.011)	1.236 (0.011)	0.074 (0.001)
$\sigma_{{e_{c \cdot 6} }}^{2}$	293 (9)	0.242 (0.008)	0.369 (0.011)	0.046 (0.002)
$\sigma_{{e_{c \cdot 7} }}^{2}$	269 (11)	0.357 (0.014)	0.382 (0.015)	0.049 (0.002)
Total variance				
$\sigma_{{P_{p} }}^{2}$	2725 (24)	1.159 (0.010)	1.264 (0.010)	0.08 (0.001)
$\sigma_{{P_{c \cdot 6}^{*} }}^{2}$	2078 (64)	0.256 (0.008)	2.586 (0.079)	0.327 (0.010)
$\sigma_{{P_{c \cdot 7}^{*} }}^{2}$	2173 (88)	0.371 (0.014)	3.063 (0.119)	0.396 (0.016)
Genetic parameters				
$h_{P}^{2}$	0.12 (0.01)	0.12 (0.01)	0.02 (< 0.01)	0.08 (0.01)
$h_{c \cdot 6}^{2}$	0.05 (0.01)	0.21 (0.06)	0.006^a^ (0.005)	0.03 (0.01)
$h_{c \cdot 7}^{2}$	0.04 (0.01)	0.15 (0.04)	0.005^a^ (0.004)	0.02 (0.01)
$r_{pc}$	0.52 (0.14)	0.47 (0.16)	0.37 (0.40)	0.56 (0.17)

The variances reported are the variances estimated by the bivariate analyses with the same trait in purebreds and crossbreds analyzed jointly

*EP* total number of eggs laid per layer between 25 and 83 weeks of age, *LNVAR* natural logarithm of the variance of the deviations between the observed phenotype and the average of the batch, *SKEW* skewness of the distribution of these deviations, *AUTO-R* lag-one autocorrelation of these deviations

$\sigma_{{a_{p} }}^{2}$: additive genetic variances for purebreds; $\sigma_{{a_{c} }}^{2}$: additive genetic variances for crossbreds; $\sigma_{{e_{p} }}^{2}$: residual variances for purebreds; $\sigma_{{e_{c \cdot n} }}^{2}$: residual variances for r crossbreds, where $n$ is the number of hens in the cage; $\sigma_{{P_{p} }}^{2}$: total variance for purebreds; $\sigma_{{P_{c \cdot n}^{*} }}^{2}$: total variance for crossbreds; $h_{P}^{2}$: heritability estimates for purebreds; $h_{c \cdot n}^{2}$: heritability estimates for crossbreds; $r_{pc}$: genetic correlation between purebred and crossbreds

^a^Indicates $h^{2}$ not significantly different from 0 (see “Estimation of variance components for the same trait between purebreds and crossbreds” section about the LRT)

For crossbred phenotypes, EP and resilience indicator traits were estimated to be less heritable than in purebreds, except for LNVAR. Although estimates of additive genetic variances were of the same magnitude for purebred and crossbred phenotypes, the adjusted phenotypic variances ($\sigma_{{P_{c}^{*} }}^{2}$) were larger in crossbreds than the phenotypic variances in purebreds ($\sigma_{{P_{p} }}^{2}$), except for LNVAR. Heritability estimates ranged from 0.04 to 0.07 for EP, from 0.13 to 0.21 for LNVAR and from 0.01 to 0.03 for AUTO-R (see Tables 2 and 3). Heritability estimates for SKEW were 0.006 and 0.005 in cages of six and seven WA crossbreds, respectively (Table 2), and were not statistically significantly different from 0 in BD crossbreds (Table 3). In summary, the resilience indicator traits showed low heritability estimates in purebreds and even lower estimates in crossbreds.

### Genetic correlations between the resilience traits

Phenotypic correlations are in Additional file 3: Tables S3–S5.

In purebreds, estimates of genetic correlations were moderately negative between LNVAR and SKEW, low between LNVAR and AUTO-R (negative or positive depending on the line), and moderately negative between SKEW and AUTO-R in both lines (Table 4).

**Table 4:** Estimates of genetic correlations between traits in purebreds with their standard error in parentheses for the WA line (below the diagonal) and the BD line (above the diagonal)

WA\BD	EP	LNVAR	SKEW	AUTO-R
EP		− 0.83 (0.04)	0.49 (0.15)	0.38 (0.11)
LNVAR	− 0.72 (0.04)		− 0.67 (0.11)	0.14 (0.10)
SKEW	− 0.05 (0.09)	− 0.46 (0.07)		− 0.55 (0.15)
AUTO-R	0.25 (0.09)	− 0.01 (0.08)	− 0.21 (0.09)	

*EP* total number of eggs laid per layer between 25 and 83 weeks of age, *LNVAR* natural logarithm of the variance of the deviations between the observed phenotype and the average of the batch, *SKEW* skewness of the distribution of these deviations, *AUTO-R* lag-one autocorrelation of these deviations

In crossbreds, the estimate of the genetic correlation between LNVAR and SKEW was − 0.67 in cages of eight BD crossbreds and was not statistically significantly different from 0 for the other crossbreds (Table 5). Estimates of the genetic correlation between LNVAR and AUTO-R ranged from 0.21 to 0.83 and was not statistically significantly different from 0 in cages of six WA crossbreds (Table 6). None of the genetic correlations between SKEW and AUTO-R were statistically significantly different from 0.

**Table 5:** Estimates of genetic correlations between traits in the WA crossbreds with their standard error in parentheses, for cages of six (below the diagonal) and seven hens (above the diagonal)

6\7 hens	EP	LNVAR	SKEW	AUTO-R
EP		− 0.82 (0.06)	0.19^a^ (0.24)	− 0.22^a^ (0.13)
LNVAR	− 0.74 (0.09)		− 0.16^a^ (0.23)	0.56 (0.10)
SKEW	− 0.38 (0.18)	− 0.18^a^ (0.28)		0.46^a^ (0.30)
AUTO-R	0.21 (0.18)	0.21^a^ (0.25)	0.34^a^ (0.39)	

*EP* total number of eggs laid per layer between 25 and 83 weeks of age, *LNVAR* natural logarithm of the variance of the deviations between the observed phenotype and the average of the batch, *SKEW* skewness of the distribution of these deviations, *AUTO-R* lag-one autocorrelation of these deviations

^a^Indicates that the estimate of the genetic correlation was not significantly different from 0 (see “Methods” section on LRT)

**Table 6:** Estimates of genetic correlations between traits in the BD crossbreds with their standard error in parentheses for cages of seven (below the diagonal) and eight hens (above the diagonal)

7\8 hens	EP	LNVAR	SKEW	AUTO-R
EP		− 0.80^b^ (0.13)	0.40 (0.27)	− 0.39^a^ (0.44)
LNVAR	− 0.54 (0.20)		− 0.67 (0.27)	0.83^b^ (0.33)
SKEW	− 0.34^a^ (0.51)	− 0.70^ab^ (0.44)		− 0.42^a^ (0.67)
AUTO-R	0.14^a^ (0.26)	0.49 (0.18)	− 0.40^a^ (0.45)	

*EP* total number of eggs laid per layer between 25 and 83 weeks of age, *LNVAR* natural logarithm of the variance of the deviations between the observed phenotype and the average of the batch, *SKEW* skewness of the distribution of these deviations; *AUTO-R* lag-one autocorrelation of these deviations

^a^Indicates that the estimate of the genetic correlation was not significantly different from 0 (see “Methods” section on LRT)

^b^Indicates that the estimate of the genetic correlation was not significantly different from 1 (see “Methods” section on LRT)

In summary, our results indicate synergetic genetic correlations between the resilience indicator traits at least in the purebreds. This suggests that including one trait as a selection criterion will lead to genetic improvements of the other traits.

### Genetic correlations between egg production and resilience indicator traits

In both purebred lines, the genetic correlations were highly negative between EP and LNVAR, from lowly negative to moderately positive between EP and SKEW, and moderately positive between EP and AUTO-R (Table 4).

In the crossbreds, genetic correlation estimates were highly negative between EP and LNVAR, ranged from − 0.38 to 0.40 between EP and SKEW (Tables 5 and 6). Although in the same direction as their respective estimates in the purebreds, the magnitude of the estimate of the genetic correlation between EP and SKEW was higher in the crossbreds. Estimates of the genetic correlations of EP with AUTO-R ranged from − 0.39 to 0.21.

In summary, estimates of genetic correlations of EP with LNVAR were in all cases negative and therefore favorable. In contrast, estimates of genetic correlations of EP with SKEW and AUTO-R were sometimes positive and sometimes negative, but never very strong.

### Purebred–crossbred genetic correlations

Estimates of genetic correlations for the same trait between purebreds and crossbreds ($r_{pc}$) ranged from 0.16 to 0.63 across traits (Tables 2 and 3). Generally speaking, the corresponding estimates of $r_{pc}$ were higher in the BD than in the WA line. All estimates of $r_{pc}$ in this study were statistically significantly different from 1, except for the estimate for SKEW, which had large standard errors. This indicates that the traits in purebreds and crossbreds (EP and the resilience indicator traits) are genetically different traits but do share part of the same genetic background.

## Discussion

Our goal was to study general resilience of layer chickens, which is expected to be related to the capacity of an animal to cope with different types of challenges: disease, climate change, negative social interactions, etc. [5, 6, 10]. Thus, we explored the genetic background of LNVAR, AUTO-R, and SKEW of the deviations of weekly egg production of an individual hen from batch average to assess their potential as selection criteria for resilience in layers. Our results indicate that these resilience indicator traits are lowly heritable and are genetically different in purebred and crossbred laying hens. Moreover, LNVAR showed favorable genetic correlations with egg production.

### Egg production

Our estimates of heritability for EP in purebreds were low and consistent with those from previous studies that were performed on EP data for similar time frames and environments (ranging from 0.01 to 0.20 across the laying period [32, 33]). In general, reported estimates of heritability for egg production traits are higher for purebreds than for crossbreds [34, 35], which appears unexpected because part of the dominance variance being captured in the additive genetic variance, hence heritability is usually higher in crossbreds than in purebreds [36]. However, estimates of residual variances vary greatly between studies and are mostly higher in crossbreds than in purebreds, which may be due to environmental conditions of crossbreds varying more than those of purebreds and makes comparisons complicated. Because purebred EP is recorded in individual cages and crossbred EP is recorded in group cages, social interactions may increase phenotypic variation for crossbred EP. Differences in heritability estimates between individual and group records have also been reported in the literature [28, 29, 37]. These differences can be explained by differences in the estimation of variance components, either because of incomplete information on the relationships between individuals due to the use of group information instead of individual information (in purebreds the complete pedigree is known while in purebred only the sire branch is known), or modelling choices (e.g. taking social interactions into account or not). Yet, when using individual data to simulate group records, estimates of the variance components based on individual and group records are the same [30].

### Using LNVAR and AUTO-R to improve resilience to disturbances

LNVAR is expected to be an indicator of both the severity and of the duration of a perturbation that the animal is experiencing, i.e. animals with a small variance are less affected by a perturbation than those with a large variance [6]. In our study, LNVAR was the most promising resilience selection criterion, because it had the highest heritability and also had a favorable genetic correlation with EP.

Our estimates of heritability for LNVAR of purebred EP (estimates of 0.11 in WA and 0.12 in BD) were similar to the estimate of heritability for LNVAR of body weight (repeated records) of layers related to the WA line (estimate of 0.10) [18]. Few studies have reported genetic parameters for LNVAR for multiple observations on one individual. LNVAR is by definition the natural logarithm of the variance of the residuals from an animal model of a trait [6]. In layers, heritabilities of 0.01 have been reported for residual variation of eggshell color at the individual egg level and of 0.15 at the level of 15 to 20 eggs measured per hen [20], and of 0.10 for residual variation of egg weight (also repeated records) [19]. In broilers, low heritabilities (lower than 0.05) have been reported for residual variation of body weight (one record per individual) [38–40]. Other studies in dairy cattle, pigs, and aquatic species, with either one record per individual or repeated records have also reported mostly low heritabilities for residual variance (ranging from 0.01 to 0.14; [16, 41–45]).

In our study, the genetic correlation of LNVAR with EP was favorable and negative (ranging from − 0.83 to − 0.54). In other species, LNVAR has also been shown to have favorable genetic correlations with other traits. In pigs, lower day-to-day variance in feed intake (root mean square error of prediction of feed intake) was genetically correlated with lower mortality and fewer health treatments [17]. In dairy cattle, LNVAR of milk yield deviations was found to be favorably genetically correlated with health (udder and hoof), longevity (productive longevity, calf and maternal survival at birth), fertility (calving interval, first to last service interval), metabolic status (ketosis resistance), and production traits (milk yield, persistency, body condition score, and feed intake) [15, 16].

To our knowledge, our study is the first to report genetic correlations of LNVAR with EP in layers. These correlations imply that selecting for a reduced LNVAR would increase EP, which is desirable. EP has been a selection criterion for many generations and, therefore, has probably contributed to indirect genetic improvement of LNVAR. Given the low heritability of EP, adding LNVAR in the breeding goal is an opportunity to improve not only resilience but also production, in spite of the low heritability of LNVAR. The next step towards the inclusion of LNVAR in a breeding program is to derive economic values or to set the targeted genetic gain. A simplified simulation study has already shown that selecting for resilience indicators has a beneficial economic impact, and even more if health traits are not included in the breeding program, because of reduced labor and treatments [6].

In summary, although selecting layers on EP already improves LNVAR, including LNVAR in breeding programs may further enhance the improvement of resilience. The absence of trade-offs makes it relatively easy to increase resilience without a loss in selection response in EP. In addition, literature on other species shows that LNVAR is favorably associated with general immunity and health [6, 45].

The resilience measure AUTO-R is expected to be informative regarding the duration of a perturbation and thus to be an indicator of an animal’s recovery capacity: close to 0 (subsequent deviations of EP are uncorrelated) for animals with high recovery rates, close to + 1 (subsequent deviations of EP are correlated) for animals with a slow recovery rate; and close to − 1 (subsequent deviations of EP are opposite) for animals showing a compensatory response to the perturbation [6]. In our study, AUTO-R had a low heritability estimate in both purebreds and in crossbreds. These heritability estimates are similar to the heritability for AUTO-R of body weight deviations in layers (value of 0.09) [18]. Similarly, in dairy cattle, the heritability for AUTO-R of milk yield deviations was estimated at 0.09 [16]. In our study, AUTO-R was estimated to be unfavorably genetically correlated with EP in purebreds, but this relationship was less clear in crossbreds. Other studies on layers have not shown a clear genetic relationship of AUTO-R with health: a non-significant genetic correlation between AUTO-R of body weight deviations and titers of natural antibody (NAbs) isotypes in serum was reported [18]. However, in dairy cattle, AUTO-R of milk yield deviations was found to be favorably genetically correlated with health, longevity, fertility, metabolic, and production traits, but at a lower level than LNVAR [16].

Before using AUTO-R as a selection criterion, its definition as a trait needs to be further discussed to reach a consensus, and its biological impact on production traits and its relationship with health needs to be understood. Knowing both the LNVAR and AUTO-R of an animal can lead to complicated interpretations: phenotypically, animals with a low LNVAR could display an AUTO-R close to 1 because they are hardly affected by perturbations, and their subsequent deviations from EP are more alike. In the present study, we found that LNVAR and AUTO-R were favorably genetically correlated in all populations but their genetic correlations were less than 1. However, if layers have genetically a high AUTO-R when faced with a severe perturbation, they will show a slow recovery. Recovery from a perturbation may be an interesting trait in the case of severe perturbations (such as a virus outbreak or a heatwave), thus it may be more appropriate to calculate AUTO-R during periods with large deviations instead of over the whole period.

In summary, selecting purebred layers on EP is expected to increase AUTO-R, which could decrease recovery rates from perturbation. Including AUTO-R in breeding programs may further enhance the improvement of resilience because it would put more emphasis on recovery rate. The trade-off between AUTO-R and EP also highlights the need to quantify economic values when considering the inclusion of AUTO-R in breeding programs.

SKEW is expected to be an indicator of both the direction (positive or negative) and severity of a perturbation that an animal is experiencing, i.e. animals with a negative SKEW experience more negative deviations than others, which is interpreted as poor resilience [6]. However, heritability estimates for SKEW were very low in the purebreds and crossbreds from both lines, similar to previous studies in layers [18] and in cattle [16], which reported that SKEW of body weight deviations and of milk yield deviations are not heritable. SKEW is known to be highly sensitive to outliers and thus to contain more noise [6]. We conclude that SKEW does not seem to be a useful resilience indicator trait when breeding for resilient laying hens. Overall, estimates of the genetic correlation of SKEW with LNVAR and AUTO-R were favorable, which means that genetic selection on LNVAR or AUTO-R is not expected to result in degraded SKEW.

### Resilience indicators in purebreds and crossbreds are genetically different

In our study, all $r_{pc}$ estimates were statistically significantly lower than 1, ranging from 0.16 to 0.63. This suggests that EP and resilience indicators traits are genetically different between purebreds and crossbreds. Our estimates of $r_{pc}$ were rather low compared to those in the literature, which range from 0.45 to 0.87 in pigs [21] and poultry [46]. Three main factors can contribute to $r_{pc}$ being lower than 1: (i) genotype-by-genotype interactions (G × G), (ii) genotype-by-environment (G × E) interactions, and (iii) differences in measurement or definition [20, 21].

First, crossbreds only share half of the genetic characteristics of each purebred parental line. Allele frequencies likely differ between purebreds and crossbreds and such differences may have direct effects on the trait (causal variants) or indirect effects (also known as G × G: dominance or epistasis), depending on the loci involved.

Second, G × E interactions can contribute to the same trait having a different genetic architecture in different environments. Purebreds and crossbreds were raised in very different environments in our study in terms of climate, management, feed, barns, disease pressure, etc. The farms where purebreds were kept have a higher level of biosecurity than regular farms because the breeding companies cannot take any risk with the population under selection. RT-farms generally belong to farmers who collaborate with breeding companies and, although their health and care practices are usually among the best ones, the layers will experience more challenges on an RT-farm than on a nucleus farm. Commercial farm environments can still be very different from RT-farm environments (cage-free systems, winter garden access, outdoor access, etc.). This diversity of environments and their susceptibility to changes (heat waves, disease outbreak, etc.) may influence the resilience performance of hens due to differences in types, severity, and duration of the perturbations. In addition, purebreds were housed individually, while crossbreds were in group cages, in which case the presence of cage mates creates positive (e.g. grooming) as well as negative (e.g. pecking) social interactions that can affect the individuals’ wellbeing and performance. As a consequence of G × E interactions, re-ranking of sires may occur. In both lines, estimates of $r_{pc}$ were lower for LNVAR than for EP or AUTO-R, which may be due to G × E interactions having a greater role in traits based on variances than in traits based on means. In trout, G × E interactions were reported to be larger for the variance of body weight than for body weight itself [47].

Third, differences in the definition or measurement of the traits may contribute to $r_{pc}$ being lower than 1. Although the definition of the variable may be the same, values may differ due to differences in the recording machines and protocols used, (e.g. housing conditions such as individual vs. group), the commitment of the collaborators, units, errors, etc., that affect the quality of the raw data. These differences can contribute to reducing the $r_{pc}$. Although the recording of the performance was the same between purebreds and crossbreds, dealing with records from individually-housed hens or group-housed hens may lead to different traits (e.g. implication of social interactions) and thus result in reduced $r_{pc}$ (theoretical derivation [20]).

All these elements can contribute to the value of recording the traits in crossbreds in a commercial environment. Breeding programs would benefit from considering both pure- and crossbred data to improve response to selection for crossbred performance in commercial environments [23, 48, 49], because it can increase the accuracy of the estimated breeding values (EBV) of purebred animals for crossbred performance.

### Longitudinal recording to genetically improve general resilience in farm animals

Our study shows that longitudinal recording offers opportunities to develop resilience indicators that are heritable. Here, resilience indicator traits are based on deviations of production performance from the undisturbed phenotype (here defined as the contemporary group average). An alternative is to estimate the undisturbed phenotype of an individual based on various modeling approaches [14, 16, 50]. Both approaches may underestimate the undisturbed phenotype, leading to underestimation of deviations and, thus, overestimation of resilience. On the one hand, if a perturbation affects the majority of the individuals in the contemporary group, the contemporary group average will be lower than the true undisturbed phenotype. On the other hand, if a perturbation affects the phenotype of an individual permanently (i.e. poor recovery), the individual’s trajectory based on observations will be lower than the undisturbed phenotype. Another approach is to estimate breeding values that allow to calculate an undisturbed trajectory (e.g. breeding values for curve parameters) [6]. Nevertheless, when modeled, a correction for the contemporary group effect is needed to account for the difference in disturbance level between contemporary groups. More research is needed to provide insight into the genetic correlations between resilience indicator traits estimated with all three approaches and which approach would best predict resilience [51].

General resilience is likely a combination of resilience of various biological functions, including food intake, digestion, growth, maintenance, reproduction, thermoregulation, immunity, behavior, etc. [5, 6, 10]. In our study, LNVAR and AUTO-R of deviations of weekly EP may capture the resilience of (re)production, i.e. the capacity of EP to be minimally affected by disturbances or to rapidly return to the undisturbed performance. This could lead to resilience of EP but not necessarily to general resilience. Thus, future studies should jointly study LNVAR of EP, LNVAR of bodyweight, and LNVAR of feed intake (e.g. cross-correlations of deviations). Longitudinal data on different traits (egg weight, body weight, and feed intake) could also be used to identify periods with a clear perturbation at the herd level [51], which would enable the study of the capacity to recover from a perturbation (i.e. AUTO-R or slope of reaction norm). Resilience indicators based on different traits could also be combined in a selection index for general resilience.

Tools are available now for phenotype recording in layers, such as electronic nests, feeders, weighing scales, cameras, microphones, RFID chips (radio-frequency identification), other embedded sensors, NIRS or MIRS (near- or mid-infrared spectroscopy), etc., which allow the concomitant high-throughput phenotyping of different traits. Based on our results and those reported in other livestock species that show the potential of resilience indicator traits for genetic improvement of resilience, especially LNVAR, further investigations in chickens are needed (i) to find the best (combination of) production traits to calculate resilience indicators, and (ii) to record available perturbation-related indicators and relate them with the resilience indicator traits.

## Conclusions

This study shows that LNVAR and AUTO-R of weekly EP are lowly heritable in purebreds as well as in crossbreds, while SKEW had very low heritability estimates. We found that these three resilience indicator traits have favorable genetic correlations with each other and with EP. Compared to purebreds, EP and the resilience indicator traits show lower heritability estimates in crossbreds, although estimates of genetic additive variance were roughly at the same level in crossbreds and purebreds. Studies on LNVAR can go forward towards its inclusion in breeding programs, i.e. by estimating economic values, and evaluating the economic and environmental impacts. AUTO-R may add complementary information, but both its definition and its joint inclusion with LNVAR in breeding programs need to be further studied. Estimates of genetic correlations between purebreds and crossbreds of production and resilience indicator traits were low in both lines, which suggests that resilience indicator traits in crossbreds are genetically different from resilience indicator traits in purebreds, which may be partly due to individual recording in the purebreds and group recording in the crossbreds. This implies that strategies that include information on crossbreds in breeding programs would be beneficial to the genetic improvement of resilience in crossbreds.

## Supplementary Information

**Additional file 1: Table S1.** Distribution of hens from the WA line according to country and farms. **Table S2.** Distribution of hens from the BD line according to country and farms.

**Additional file 2.** Heritability estimated with pooled records of half-sibs, for regular and resilience indicator traits [28, 30, 52].

**Additional file 3: Table S3.** Phenotypic correlations among traits in purebreds, and their standard error below in italic characters, for the WA line (below the diagonal) and the BD line (above the diagonal). **Table S4.** Phenotypic correlations among traits in the WA crossbreds, and their standard error below in italic characters, for cages of 6 hens (below the diagonal) and cages of 7 hens (above the diagonal). **Table S5.** Phenotypic correlations among traits in the BD crossbreds, and their standard error below in italic characters, for cages of 7 hens (below the diagonal) and cages of 8 hens (above the diagonal).

## Data Availability

The data that support the findings of this study are available from Hendrix Genetics but restrictions apply to the availability of these data, which were used under license for the current study, and thus are not publicly available. Data are however available from the authors upon reasonable request and with permission of Hendrix Genetics (katrijn.peeters@hendrix-genetics.com).
