# Supplementary material for: Using egg production longitudinal recording to study the genetic background of resilience in purebred and crossbred laying hens
Source: Genet Sel Evol. 2022 Apr 20;54:26. doi: 10.1186/s12711-022-00716-8 (PMC9020098; doi:10.1186/s12711-022-00716-8)
Supplement: Supplementary file 1 — Additional file 1: Table S1. Distribution of hens from the WA line according to country and farms. Table S2. Distribution of hens from the BD line according to country and farms. [file 12711_2022_716_MOESM1_ESM.pdf]

Additional File 1: Further detail about the phenotypic data structure

Using egg production longitudinal recording to study the genetic background of resilience in purebred and crossbred laying hens

Nicolas Bedere[1*], Tom V.L. Berghof[2,3], Katrijn Peeters[4], Marie-Helene Pinard-Van der Laan[5], Jeroen Visscher[4], Ingrid David[6], Han A. Mulder[2]

[1]PEGASE, INRAE, Institut Agro, 35590, Saint Gilles, France

[2]Wageningen University & Research Animal Breeding & Genomics, P.O. Box 338, 6700 AH Wageningen, The Netherlands

[3]Reproductive Biotechnology, TUM School of Life Sciences, Technical University of Munich, Liesel-Beckmann-Strasse 1, 85354 Freising, Germany

[4]Hendrix Genetics B.V., P.O. Box 114, 5830 AC Boxmeer, The Netherlands

[5]Université Paris-Saclay, INRAE, AgroParisTech, GABI, 78350, Jouy-en-Josas, France

[6]GenPhySE, Université de Toulouse, INRAE, ENVT, Castanet Tolosan, France

*Corresponding author: nicolas.bedere@inrae.fr

This supplementary material provides details about the distribution of hens and cages among lines, farms, and batches.

Table S1 Distribution of hens in the WA line according to country and farms.

Line	Country	Location	Batch	Number of cages	Number of hens
WA	NL	nucleus	2012.1		2388
WA	NL	nucleus	2012.2		2147
WA	NL	nucleus	2013.1		2587
WA	NL	nucleus	2013.2		2094
WA	NL	nucleus	2013.3		2592
WA	NL	nucleus	2014.1		2372
WA	NL	nucleus	2014.2		2593
WA	NL	nucleus	2015.1		2201
WA	NL	nucleus	2015.2		2596
WA	NL	nucleus	2016.1		2368
WA	NL	nucleus	2016.2		2796
WA	NL	nucleus	2016.3		2301
WA	NL	nucleus	2017.1		3021
WA	CA	RT_farm_1	2012.1	301	2106
WA	CA	RT_farm_1	2013.2	945	6582
WA	CA	RT_farm_1	2014.2	732	5100
WA	CA	RT_farm_2	2012.2	1102	6612
WA	CA	RT_farm_3	2015.1	946	5676
WA	CA	RT_farm_4	2013.3	953	5718
WA	CA	RT_farm_4	2016.2	883	5301
WA	NL	RT_farm_5	2013.1	917	6547
WA	NL	RT_farm_5	2015.2	814	5677
WA	NL	RT_farm_6	2016.1	744	4616
WA	NL	RT_farm_7	2014.2	854	5299
WA	NL	RT_farm_7	2016.2	957	5862
WA	NL	RT_farm_8	2012.1	10	69
WA	NL	RT_farm_8	2015.1	8	56
WA	NL	RT_farm_9	2013.2	855	5963
WA	NL	RT_farm_10	2012.2	894	6223
WA	NL	RT_farm_10	2013.3	917	6403

Table S2 Distribution of hens in the BD line according to country and farms.

Line	Country	Location	Batch	Number of cages	Number of hens
BD	FR	nucleus	2012.1		2200
BD	FR	nucleus	2012.2		2243
BD	FR	nucleus	2013.1		2474
BD	FR	nucleus	2013.2		2409
BD	FR	nucleus	2013.3		2508
BD	FR	nucleus	2014.1		2471
BD	FR	nucleus	2014.2		2476
BD	FR	nucleus	2015.1		2481
BD	FR	nucleus	2015.2		2472
BD	FR	nucleus	2016.1		2475
BD	FR	nucleus	2016.2		2446
BD	FR	nucleus	2016.3		2472
BD	FR	nucleus	2017.1		2476
BD	CA	RT_farm_11	2013.1	427	3391
BD	CA	RT_farm_11	2014.1	389	3099
BD	NL	RT_farm_12	2012.1	64	448
BD	NL	RT_farm_13	2012.2	518	4123
BD	NL	RT_farm_14	2012.1	328	2344
BD	NL	RT_farm_15	2011.1	525	3687
BD	NL	RT_farm_16	2012.2	685	4885
BD	NL	RT_farm_16	2015.1	434	3040
BD	NL	RT_farm_16	2016.2	528	3739
