# Supplementary material for: Using egg production longitudinal recording to study the genetic background of resilience in purebred and crossbred laying hens
Source: Genet Sel Evol. 2022 Apr 20;54:26. doi: 10.1186/s12711-022-00716-8 (PMC9020098; doi:10.1186/s12711-022-00716-8)
Supplement: Supplementary file 2 — Additional file 2. Heritability estimated with pooled records of half-sibs, for regular and resilience indicator traits [28, 30, 52]. [file 12711_2022_716_MOESM2_ESM.pdf]

Additional File 2: Estimating heritability with pooled records

---

Using egg production longitudinal recording to study the genetic background of resilience in purebred and crossbred laying hens

Nicolas Bedere[1*], Tom V.L. Berghof[2,3], Katrijn Peeters[4], Marie-Helene Pinard-Van der Laan[5], Jeroen Visscher[4], Ingrid David[6], Han A. Mulder[2]

[1]PEGASE, INRAE, Institut Agro, 35590, Saint Gilles, France

[2]Wageningen University & Research Animal Breeding & Genomics, P.O. Box 338, 6700 AH Wageningen, The Netherlands

[3]Reproductive Biotechnology, TUM School of Life Sciences, Technical University of Munich, Liesel-Beckmann-Strasse 1, 85354 Freising, Germany

[4]Hendrix Genetics B.V., P.O. Box 114, 5830 AC Boxmeer, The Netherlands

[5]Université Paris-Saclay, INRAE, AgroParisTech, GABI, 78350, Jouy-en-Josas, France

[6]GenPhySE, Université de Toulouse, INRAE, ENVT, Castanet Tolosan, France

*Corresponding author: nicolas.bedere@inrae.fr

This supplementary material aims to explain in more detail the scaling of the estimation of heritability with pooled records of half-sibs [25,27]. For crossbreds, the heritability was calculated, per cage size, as:

$$h^{2^*} = \frac{4 \times \sigma_s^2}{\sigma_s^2 + n \times \sigma_{e^*}^2}$$

where n was the number of hens grouped in the cage, $4 \times \sigma_s^2 = \sigma_a^2$ was the additive genetic variance for crossbreds; $\sigma_{e^*}^2$ was the residual variance for crossbreds.

1. Explaining the multiplicative term "n" for the residuals

A phenotype (P) of animal i can be decomposed by the following formulae:

$$P_i = \mu + a_i + e_i \quad (1)$$

where a_i is the breeding value of animal i, with $\mathbf{a}{\sim}N(0, \mathbf{A}\sigma_a^2)$ where **A** is the genetic relationship matrix, and e_i the environmental effect (or residual) with $\mathbf{e}{\sim}N(0, \mathbf{I}\sigma_e^2)$ where **I** is the identity matrix. For animal i born from sire j and dam k, $a_i = \frac{1}{2}a_j + \frac{1}{2}a_k + MS_i$ where MS_i is the Mendelian sampling (random error due to meiosis) with variance $\sigma_{MS}^2 = \frac{1}{2}\sigma_a^2$. The heritability of the P_i is:

$$h^2 = \frac{\sigma_a^2}{\sigma_a^2 + \sigma_e^2}$$

If the observed phenotype is a pooled average from sire j half-sibs, $P^*{}_j = \frac{\sum_{i=1}^{n} P_i}{n}$, then:

$$P^*{}_j = \mu + \frac{1}{2}a_j + \frac{\sum_{i=1}^{n}\frac{1}{2}a_{d_i} + \sum_{i=1}^{n} MS_i + \sum_{i=1}^{n} e_i}{n}$$

Where d_i is the index for the dam of animal i, thus applying a sire model on pooled records:

$$P_j^* = \mu + s_j + e_j^* \ (2)$$

Where $s_j = \frac{1}{2}a_j$ and $e_j^* = \frac{1}{n}\sum_{i=1}^{n}\left(\frac{1}{2}a_{di} + MS_i + e_i\right)$

Variance components estimated in model (2) are thus:

$\sigma_s^2 = \frac{\sigma_a^2}{4}$ and $\sigma_{e^*}^2 = \frac{1}{n^2}\left(\frac{n}{4}\sigma_a^2 + \frac{n}{2}\sigma_a^2 + n\sigma_e^2\right) = \frac{1}{n}\left(\frac{3}{4}\sigma_a^2 + \sigma_e^2\right)$

The heritability of the trait from variance components estimated in model (2) should then computed as:

$$h^{2^*} = \frac{4\sigma_s^2}{\sigma_s^2 + n\sigma_{e^*}^2}$$

$$\Leftrightarrow h^{2^*} = \frac{\sigma_a^2}{\frac{\sigma_a^2}{4} + \frac{3}{4}\sigma_a^2 + \sigma_e^2}$$

$$\Leftrightarrow h^{2^*} = \frac{\sigma_a^2}{\sigma_a^2 + \sigma_e^2} = h^2$$

$$QED$$

More information and validation with simulated situations can be found in the discussion section of Katrijn Peeters' Ph.D. thesis [27].

2. The calculation of heritability for pooled resilience indicator traits, that are based on deviations

In the following subsections, the subscripts used are:

Subscript	Standing for
i	individual
j	sire
t	time
g	group
m	number of records (times)
n	number of individuals in the group

Other notations are:

EP_{it} is the eggs production of animal i at time t, $EP_{it} = \mu_t + a_i + e_{it}$

μ_t is the flock's weekly average EP,

$Dev_{it} = EP_{it} - \mu_t = a_i + e_{it}$ is the deviation of animal i at time t,

$\overline{Dev_i} = \frac{1}{m}\sum_{t=1}^{m} Dev_{it}$ is the average deviation for animal i,

$Dev_{it} - \overline{Dev_i} = e_{it}$,

$\sigma^2_{Dev,i} = \frac{\sum_{t=1}^{m}(Dev_{it} - \overline{Dev_i})^2}{m-1} = \frac{\sum_{t=1}^{m}(e_{it})^2}{m-1}$ is the variance of the deviation for animal i,

For pooled records, let's consider a group g of n half-sibs of sire j:

$EP^*_{jt} = \frac{\sum_{i\in g} EP_{it}}{n}$ is the egg production calculated as pooled records for sire j at time t,
And: $EP^*_{jt} = \mu_t + \frac{1}{n}\left(\sum_{i\in g} a_i + \sum_{i\in g} e_{it}\right)$
Thus: $EP^*_{jt} = \mu_t + \frac{1}{2}a_j + \frac{1}{n}\sum_{i\in g}\left(\frac{1}{2}a_{di} + MS_i + e_{it}\right)$,

$Dev^*_{jt} = EP^*_{jt} - \mu_t$ is the deviation for sire j at time t,

$\overline{Dev^*_j}$ is the average deviation for sire j,
$\overline{Dev^*_j} = \frac{1}{m}\sum_{t=1}^{m} Dev^*_{jt} = \frac{1}{2}a_j + \frac{1}{n}\sum_{i=1}^{n}\left(\frac{1}{2}a_{di} + MS_i\right) + \frac{1}{nm}\sum_{t=1}^{m}\sum_{i\in g} e_{it}$,

$Dev^*_{jt} - \overline{Dev^*_j} = \frac{\sum_{i\in g} e_{it}}{n}$

We note $\sigma^2_{Dev*,j}$ the variance of the deviation for sire j based on pooled records

2.1. LNVAR

Let's consider repeated observations on animals, recorded at the individual scale and as pooled records of half-sibs groups. Traits are defined as:

<table>
<tr><th>$\boldsymbol{LNVAR_{ind}}$</th><th>$\boldsymbol{LNVAR_{pool}}$</th></tr>
<tr><td>$LNVAR_{ind,i} = ln(\sigma^2_{Dev,i}) = ln\left(\frac{\sum_{t=1}^{m}(e_{it})^2}{m-1}\right)$</td><td>$LNVAR_{pool,j} = ln(\sigma^2_{Dev*,j})$

The relationship between individual and pooled records can be approximated as:

$LNVAR_{pool,j} = ln\left(\left[\frac{1+(n-1)(¼h^2_g+c^2_g)}{n}\right]\sigma^2_{Dev,j}\right)$
$= ln\left(\left[\frac{1+(n-1)(¼h^2_g+c^2_g)}{n}\right]\right) + ln(\sigma^2_{Dev,j})$
$= K + LNVAR_{ind,j}$

where $(¼h^2_g + c^2_g)$ is the resemblance of half-sib cage mates due to genetics and a possible common cage effects (c^2_g). h^2_g is the heritability of $\sigma^2_{Dev,j}$ (prior to the ln-transformation).

This shows that $LNVAR_{pool} \neq \overline{LNVAR_{ind}}$

The trait analyzed based on pooled records ($LNVAR_{pool}$) differs from the trait analysed based on individual records ($LNVAR_{ind}$) with a factor K. The magnitude of K depends upon n (number of cage mates), the heritability of the trait (h^2_g) and the presence of common environmental (group) effects (c^2_g).</td></tr>
<tr><th>$\boldsymbol{estimating\ h^2\ for\ LNVAR_{ind}}$</th><th>$\boldsymbol{estimating\ h^2\ for\ LNVAR_{pool}}$</th></tr>
<tr><td>Animal model:
$LNVAR_{ind_i} = \mu_{LN} + a_{LNi} + e_{LNi}$
thus:
$$h^2_{LNVARind} = \frac{\sigma^2_{aLN}}{\sigma^2_{aLN} + \sigma^2_{eLN}}$$</td><td>Sire model:
$LNVAR_{pool_{jg}} = \mu_{LNp} + s_{LNpj} + e_{LNpjg}$
As a constant, K will not contribute to variation but to the overall mean μ_{LNp}.
$$h^2_{LNVARpool} = \frac{4\sigma^2_{sLNp}}{\sigma^2_{sLNp} + \sigma^2_{eLNp}}$$</td></tr>
</table>

2.2. AUTO-R

Let's consider repeated observations on animals, recorded at the individual scale and as pooled records of half-sibs groups. Traits are defined as:

$\boldsymbol{AUTOR_{ind}}$	$\boldsymbol{AUTOR_{pool}}$
$AUTOR_{ind,i} = \frac{cov(e_{it}, e_{it+1})}{\sigma^2_{ei}}$<br>$AUTOR_{ind,i} = \frac{\sum_{t=1}^{m-1}(Dev_{it}-\overline{Dev_i})(Dev_{it+1}-\overline{Dev_i})}{\sum_{t=1}^{m}(Dev_{it}-\overline{Dev_i})^2}$	$AUTOR_{pool,j} = \frac{cov\left(\frac{\sum_{i\in g} e_{it}}{n}, \frac{\sum_{i\in g} e_{it+1}}{n}\right)}{var\left(\frac{\sum_{i\in g} e_{it}}{n}\right)}$<br>With a homogeneous residual variance σ^2_e for half-sibs:<br>$AUTOR_{pool,j} = \frac{\frac{1}{n^2}\sum_{i\in g} cov(e_{it}, e_{it+1})}{\frac{\sigma^2_e}{n}}$<br>$= \frac{\sum_{i\in g} cov(e_{it}, e_{it+1})}{n\sigma^2_e}$<br>$= \frac{1}{n}\sum_{i\epsilon g} AUTOR_{ind} = \overline{AUTOR_{ind}}$<br>Meaning that calculating AUTO-R on the pool performance of a group (mean) is identical to the mean of the individual AUTO-R of the group.
$\boldsymbol{estimating\ h^2\ for\ AUTOR_{ind}}$	$\boldsymbol{estimating\ h^2\ for\ AUTOR_{pool}}$
Animal model:<br>$AUTOR_{ind_i} = \mu_{AC} + a_{ACi} + e_{ACi}$<br>thus:<br>$h^2_{ACind} = \frac{\sigma^2_{aAC}}{\sigma^2_{aAC} + \sigma^2_{eAC}}$	Sire model:<br>$AUTOR_{pool_{jg}} = \mu_{ACp} + s_{ACpj} + e_{ACpjg}$<br>Given that<br>$AUTOR_{pool} = \overline{AUTOR_{indiv}}$<br>$h^2_{ACpool} = \frac{4\sigma^2_{sACp}}{\sigma^2_{sACp} + n\sigma^2_{ACp}}$<br>As explained in section 1

2.3. SKEW

Let's consider repeated observations on animals, recorded at the individual scale and as pooled records of half-sibs groups. Traits are defined as:

$\boldsymbol{P = SKEW_{ind}}$	$\boldsymbol{P^* = SKEW_{pool}}$
$SKEW_{ind,i}$<br>$= \frac{m}{(m-1)(m-2)} \sum_{t=1}^{m} \left(\frac{Dev_{it} - \overline{Dev_i}}{\sigma_e}\right)^3$<br>$= M \sum_{t=1}^{m} \left(\frac{e_{it}}{\sigma_e}\right)^3$<br>where:<br>$M = \frac{m}{(m-1)(m-2)}$	$SKEW_{pool,j} = SKEW\left(\frac{\sum_{i\in g} e_{it}}{n}\right) = SKEW\left(\sum_{i\in g} e_{it}\right)$<br>According to Eriksson (p.29, eq.20) [52] for the skewness of n independent variables<br>$SKEW\left(\sum_{i\in g} e_{it}\right) = \frac{\sum_{i\in g} \mu_{3i}}{\left(\sum_{i\in g} \sigma_{e_i}^2\right)^{3/2}}$<br>Where $\mu_{3i} = \frac{1}{m}\sum_{t=1}^{m} e_{ij}{}^3$<br>With the same residual variance for all half-sibs in group g, $(\sigma_{e_i}^2 = \sigma_e^2)$, we obtain:<br>$SKEW\left(\sum_{i\in g} e_{it}\right) = \frac{\sum_{i\in g} \mu_{3i}}{(n\times\sigma_e^2)^{\frac{3}{2}}}$<br>$SKEW\left(\sum_{i\in g} e_{it}\right) = \frac{1}{n^{3/2}} \frac{\sum_{i\in g} \mu_{3i}}{(\sigma_e^2)^{\frac{3}{2}}}$<br>$SKEW\left(\sum_{i\in g} e_{it}\right) = \frac{1}{\sqrt{n}} \times \frac{\sum_{i\in g} SKEW_{ind,i}}{n}$<br>Thus the relationship between individual and polled records is:<br>$SKEW_{pool,j} = \frac{1}{\sqrt{n}} \times \overline{SKEW_{ind,i}}$<br>Meaning that calculating SKEW on the pool performance of a group (mean) is identical to the mean of the individual SKEW of the group.<br>The multiplicative term $\frac{1}{\sqrt{n}}$ is a characteristics of skewness: due to the central limit theorem, large n leads to a symmetric normal distribution (non-skewed).

$\boldsymbol{estimating\ h^2\ for\ SKEW_{ind}}$	$\boldsymbol{estimating\ h^2\ for\ SKEW_{pool}}$
Animal model:<br>$SKEW_{ind_i} = \mu_{SK} + a_{SKi} + e_{SKi}$<br>thus:<br>$$h^2_{SKind} = \frac{\sigma^2_{aSK}}{\sigma^2_{aSK} + \sigma^2_{eSK}}$$	Sire model:<br>$SKEW_{pool_{jg}} = \mu_{SKp} + s_{SKpj} + e_{SKpjg}$<br>Given that $SKEW_{pool_{jg}} = \frac{1}{\sqrt{n}} \times \overline{SKEW_{ind}}$,<br>$$h^2_{SKpool} = \frac{4\sigma^2_{sSKp}}{\sigma^2_{sSKp} + n\sigma^2_{eSKp}}$$<br>As explained in section 1
