# Supplementary material for: Using egg production longitudinal recording to study the genetic background of resilience in purebred and crossbred laying hens
Source: Genet Sel Evol. 2022 Apr 20;54:26. doi: 10.1186/s12711-022-00716-8 (PMC9020098; doi:10.1186/s12711-022-00716-8)
Supplement: Supplementary file 3 — Additional file 3: Table S3. Phenotypic correlations among traits in purebreds, and their standard error below in italic characters, for the WA line (below the diagonal) and the BD line (above the diagonal). Table S4. Phenotypic correlations among traits in the WA crossbreds, and their standard error below in italic characters, for cages of 6 hens (below the diagonal) and cages of 7 hens (above the diagonal). Table S5. Phenotypic correlations among traits in the BD crossbreds, and their standard error below in italic characters, for cages of 7 hens (below the diagonal) and cages of 8 hens (above the diagonal). [file 12711_2022_716_MOESM3_ESM.pdf]

Additional File 3: Phenotypic correlations

Using egg production longitudinal recording to study the genetic background of resilience in purebred and crossbred laying hens

Nicolas Bedere[1*], Tom V.L. Berghof[2,3], Katrijn Peeters[4], Marie-Helene Pinard-Van der Laan[5], Jeroen Visscher[4], Ingrid David[6], Han A. Mulder[2]

[1]PEGASE, INRAE, Institut Agro, 35590, Saint Gilles, France

[2]Wageningen University & Research Animal Breeding & Genomics, P.O. Box 338, 6700 AH Wageningen, The Netherlands

[3]Reproductive Biotechnology, TUM School of Life Sciences, Technical University of Munich, Liesel-Beckmann-Strasse 1, 85354 Freising, Germany

[4]Hendrix Genetics B.V., P.O. Box 114, 5830 AC Boxmeer, The Netherlands

[5]Université Paris-Saclay, INRAE, AgroParisTech, GABI, 78350, Jouy-en-Josas, France

[6]GenPhySE, Université de Toulouse, INRAE, ENVT, Castanet Tolosan, France

*Corresponding author: nicolas.bedere@inrae.fr

This supplementary material aims to provide the reader with the phenotypic correlations, calculated in ASReml in order to account for the other fixed and random effects of the model.

Table S3 Phenotypic correlations among traits in purebreds, and their standard error in italic bellow, for the **WA** line (below the diagonal) and the **BD** line (above the diagonal)

WA \ BD	EP	LNVAR	SKEW	AUTO-R
**EP**		-0.59<br>*<0.01*	-0.21<br>*0.01*	-0.35<br>*0.01*
**LNVAR**	-0.58<br>*<0.01*		-0.32<br>*0.01*	0.51<br>*0.01*
**SKEW**	-0.15<br>*0.01*	-0.33<br>*0.01*		0.04<br>*0.01*
**AUTO-R**	-0.37<br>*0.01*	0.42<br>*0.01*	0.03<br>*0.01*	

The abbreviations of the traits stand for the total number of eggs laid per layer between 25 and 83 weeks of age (EP), the natural logarithm of the variance of the deviations between the observed phenotype and the average of the flock (LNVAR); skewness of the distribution of these deviations (SKEW), the lag-one autocorrelation of these deviations (AUTO-R).

Table S4 Phenotypic correlations among traits in the **WA** crossbreds, and their standard error in italic bellow, for cages of 6 hens (below the diagonal) and cages of 7 hens (above the diagonal)

6\7	EP	LNVAR	SKEW	AUTO-R
**EP**		-0.59<br>*0.01*	<0.01<br>*0.01*	-0.39<br>*0.01*
**LNVAR**	-0.51<br>*0.01*		-0.29<br>*0.01*	0.67<br>*0.01*
**SKEW**	<0.01<br>*0.01*	-0.32<br>*0.01*		-0.19<br>*0.01*
**AUTO-R**	-0.31<br>*0.01*	0.67<br>*0.01*	-0.22<br>*0.01*	

The abbreviations of the traits stand for the total number of eggs laid per layer between 25 and 83 weeks of age (EP), the natural logarithm of the variance of the deviations between the observed phenotype and the average of the flock (LNVAR); skewness of the distribution of these deviations (SKEW), the lag-one autocorrelation of these deviations (AUTO-R).

Table S5 Phenotypic correlations among traits in the **BD** crossbreds, and their standard error in italic bellow, for cages of 7 hens (below the diagonal) and cages of 8 hens (above the diagonal)

7\8	EP	LNVAR	SKEW	AUTO-R
**EP**		-0.51<br>*0.02*	0.12<br>*0.03*	-0.29<br>*0.03*
**LNVAR**	-0.51<br>*0.02*		-0.33<br>*0.03*	0.70<br>*0.01*
**SKEW**	0.13<br>*0.02*	-0.34<br>*0.02*		-0.12<br>*0.03*
**AUTO-R**	-0.27<br>*0.02*	0.58<br>*0.01*	-0.13<br>*0.02*	

The abbreviations of the traits stand for the total number of eggs laid per layer between 25 and 83 weeks of age (EP), the natural logarithm of the variance of the deviations between the observed phenotype and the average of the flock (LNVAR); skewness of the distribution of these deviations (SKEW), the lag-one autocorrelation of these deviations (AUTO-R).
